# Breast Cancer and Cardiovascular Risk: The Role of Dyslipidemia, Inflammation and Obesity

**DOI:** 10.3390/diagnostics16020308

**Published:** 2026-01-18

**Authors:** Barbara Loboda, Darko Zdravkovic, Nebojsa Ivanovic, Natasa Colakovic, Simona Petricevic, Milan Gojgic, Bogdan Crnokrak, Vladimir Milosavljevic, Viseslav Popadic, Dragana Bjelica, Visnja Stojanovic, Marija Zdravković

**Affiliations:** 1 University Hospital Medical Center “Bežanijska kosa”, 11071 Belgrade, Serbiabcrnokrak@yahoo.com (B.C.); viseslavpopadic@gmail.com (V.P.);; 2 Faculty of Medicine, University of Belgrade, 11000 Belgrade, Serbia

**Keywords:** breast cancer, metabolic syndrome, inflammation, dyslipidemia, obesity, diabetes

## Abstract

Cardiovascular diseases, malignancy, and diabetes mellitus are the most common chronic non-communicable diseases affecting the population in Serbia. According to The Cancer Registry of the Republic of Serbia, breast cancer is the most common cancer affecting women in Serbia. Every year, 4600 women get diagnosed with BC, and 1600 women die from this disease. Every eighth woman in Serbia is diagnosed with BC. This review aims to summarize clinical and theoretical information about breast cancer, metabolic syndrome and cardiovascular risk connection. The literature search was conducted through PubMed, Google Scholar and cross-references in January 2024. We concluded that although there is a well-established connection between cardiovascular risk, metabolic syndrome, inflammation, dyslipidemia, obesity, diabetes mellitus and breast cancer, more multicenter prospective clinical studies are needed to establish the precise association and pathophysiological mechanisms.

## 1. Introduction

Cardiovascular diseases (CVD), malignancy, and diabetes mellitus (DM) are the most common chronic non-communicable diseases affecting the population in Serbia. According to The Cancer Registry of the Republic of Serbia, breast cancer (BC) is the most common cancer affecting women in Serbia. Every year, 4600 women get diagnosed with BC, and 1600 women die from this disease. Every eighth woman in Serbia is diagnosed with BC [1].

In 2022, 51,624 people died of cardiovascular disease in Serbia (23,695 male, 27,929 female). Cardiovascular diseases account for 47.3% of all causes of death and are the leading cause of death in Serbia. Women (54.1%) die more often from this cause than men (45.9%). The most common CVDs are ischemic heart disease and cerebrovascular disease [1].

Diabetes mellitus (DM) is one of the most common chronic diseases worldwide. The number of individuals affected by DM is steadily increasing and is reaching the proportions of a global pandemic. According to the World Health Organization (WHO), approximately 422 million people worldwide have diabetes. Among all diabetes patients, the majority are affected by diabetes mellitus type 2 (DM2) (95%). Diabetes is the fifth leading cause of death globally and the third leading cause of death in Serbia among all mortality causes. Approximately 3000 individuals die annually in our country due to diabetes-related complications [1].

Breast cancer is a heterogeneous disease with multiple subtypes. There are several classifications of BC. The fundamental classification involves histological categorization of BC, wherein the most common is invasive ductal carcinoma, representing 70–80% of all invasive carcinomas, followed by invasive lobular carcinoma at 10%, with the remainder comprising rarer forms such as mucinous, cribriform, papillary, tubular, medullary, metaplastic, and apocrine types [2]. The most commonly used and widely employed classification is the immunohistochemical classification, which defines molecular subtypes of BC. Immunohistochemistry assesses the presence of estrogen receptors (ER), progesterone receptors, and human epidermal growth factor 2. Based on the expression of these receptors, breast cancer is classified into the following subtypes: Luminal A, Luminal B, HER2-positive, and triple-negative. The molecular classification of BC holds significant clinical importance as different subtypes exhibit distinct characteristics and prognoses, thereby guiding oncological treatment based on molecular subtypes [3].

Epidemiological studies have shown that factors such as early menarche, postmenopausal weight gain, a high-fat diet, and long-term use of estrogen therapy are related to a high risk of BC [4,5]. Recent studies have shown that a lifestyle characterized by reduced physical activity and a diet rich in fat, refined carbohydrates, and animal proteins lead to a metabolic syndrome, which is very important in BC initiation [6,7].

Metabolic syndrome (MetS) represents a group of risk factors for CVD and DM and is a growing public health problem. These risk factors are obesity (mostly centripetal obesity), disorders of glycemia, high blood pressure, and dyslipidemia [8].

Recent clinical, experimental, and epidemiologic studies support the hypothesis that MetS could be a significant etiologic factor for the onset and progression of malignant tumors, including BC [9].

Many studies have shown that inflammation is present in cancer and is associated with its development and progression. It is also proposed that some chronic diseases, such as diabetes and obesity, increase chronic inflammation [10].

This systematic review will summarize clinical and theoretical information about the BC, MetS, inflammation, dyslipidemia, obesity, diabetes, and cardiovascular risk connection.

## 2. Materials and Methods

We conducted a literature search through PubMed, Google Scholar and cross-references in January 2024. The search consisted of the terms “breast cancer,” “metabolic syndrome,” “obesity,” “dyslipidemia,” “diabetes,” and “inflammation.” Case reports and case series were excluded from the search. Only full-text articles written in English were included.

## 3. Discussion

### 3.1. Metabolic Syndrome

The metabolic syndrome represents a cluster of risk factors that lead to the onset of CVD and type 2 diabetes. These factors are visceral obesity, insulin resistance, hypertension, low levels of serum HDL cholesterol, and high levels of serum triglycerides. MetS exists when three or more factors are present [11]. The results of previous epidemiological, experimental, and clinical studies support the hypothesis that MetS may be a significant etiological factor in the development and progression of certain malignancies [9]. Several recent studies have demonstrated an association between MetS and BC [12].

There is a presumption that the association of obesity and MetS increases the risk of developing diseases, including cancer.

Studies have shown that obesity manifests in several phenotypes regarding its association with MetS. This association implies that individuals with normal body weight but some abnormal metabolic characteristics such as central obesity, glucose intolerance, elevated blood pressure, or dyslipidemia are classified as metabolically unhealthy, thereby increasing the risk of CVD and all diseases for which MetS is a risk factor [13,14]. The molecular mechanisms leading to this association are presumed to be impaired insulin sensitivity, proinflammatory markers, and oxidative stress [15,16].

The following phenotypes have been defined: metabolically healthy normal weight (MHNW), metabolically unhealthy normal weight (MUNW), metabolically healthy overweight/obese (MHO), metabolically unhealthy overweight/obese (MUO), and sarcopenic obese (SO). Most commonly, comparisons are made between MHO and MUO individuals, as they have a BMI greater than 25 kg/m^2^ but significant differences in metabolic profile [17].

The study conducted by Mark J. Gunter et al. in 2015 [18] investigated the risk of BC development in (MHO) postmenopausal women. They compared the risk of BC in MHNW women with MUO, MHO, and MUNW women. MUO women had a significantly elevated risk of BC compared to MHNW women (*p* = 0.005). A similar association was identified for MUNW women, but this association did not reach statistical significance (*p* = 0.11). No association was observed between MHO women and BC occurrence (*p* = 0.83). Furthermore, there was a statistically significant difference in the risk of BC occurrence between MHO and MUO women (*p* < 0.0001). MHO women did not have an increased risk of breast cancer occurrence compared to MHNW women (*p* = 0.82). The research showed that MUO women are at a significantly higher risk of BC compared to MHO women (*p* < 0.0001). Findings from this study are consistent with previous results, indicating that individuals with excess weight who are metabolically healthy (normal insulin resistance, normal lipid status) are not at an increased risk for cardiovascular and other diseases associated with metabolic syndrome. Research has demonstrated that hyperinsulinemia is an independent risk factor for BC occurrence [18].

In a prospective cohort study conducted by Yong-Moon Mark Park et al. in 2017 [19], the results showed that individuals with the MUO phenotype have a higher risk of breast cancer compared to the MHNW phenotype. Women with MUNW or MHO phenotypes had a similarly increased risk of BC after menopause compared to MHNW. The study results indicated that the MUNW phenotype carries an increased risk of BC occurrence in postmenopausal women, while overweight/obese women have an elevated risk of BC regardless of metabolic status, which differs from the findings of the study by Gunter et al. [18,19].

The discussion noted the controversy of whether MHO is truly a phenotype without disease risk. A meta-analysis conducted by Zheng et al. in 2016 showed that the MHO phenotype is not associated with an increased risk of all-cause mortality but has an elevated risk for CVD compared to the MHNW phenotype [20]. In contrast to the findings of the meta-analysis mentioned above by Zheng et al., a meta-analysis conducted by Eckel et al. in 2016 showed that the MHO phenotype is associated with a lower risk of CVD than MUNW and MUO phenotypes [21].

Further research found that such results of meta-analyses that are not in correlation may be because of the instability of the MHO phenotype, which, due to obesity, may progress into the MUO phenotype. Therefore, obesity can be an independent factor for postmenopausal BC [22].

The prospective study conducted by Lynn Moore et al. in 2014 [23] examined whether metabolic health reduced the risk of obesity-related cancer in adults in the Framingham study and showed that overweight adults with elevated glucose levels had a statistically significant two-fold increased risk of developing obesity-related cancer. In contrast, overweight adults with normal glucose had a 50% increased risk. The study also showed that overweight women with elevated blood glucose had a 2.6-fold increased risk of female reproductive cancers (cervical, endometrial, and uterine) and postmenopausal breast cancer, while overweight women with normal glucose levels had a 70% increased risk. In each risk category, the risk was the highest for the metabolically unhealthy obese (MUO) individuals, while metabolically healthy obese (MHO) individuals had intermediate risk. In this study, there was no evidence that metabolic disorders in the absence of overweight/obesity increase the risk of developing these types of cancers [23]. Results from the studies discussed in this paragraph are presented in Table 1.

### 3.2. Inflammation

Since the late 19th century, a theory has suggested a link between inflammation and tumors. Initially, the presence of cells typical for inflammation was observed in histopathological specimens [24]. Many studies have successfully demonstrated the association between inflammation and the development or progression of certain malignant tumors such as colorectal cancer, esophageal cancer, hepatocellular cancer, bladder cancer, gastric cancer, lung cancer, and others. Numerous studies have investigated the association between inflammation and BC, but this role remains incompletely understood [25].

Epidemiological studies have shown that chronic inflammation may predispose to the development of certain types of tumors [23]. There are several causes of chronic inflammation, including microbial infections (e.g., Helicobacter pylori infection in gastric cancer and lymphoma), autoimmune diseases (e.g., the association of inflammatory bowel disease with colon cancer), and inflammatory conditions of unknown origin (e.g., the association of prostatitis with prostate cancer) [26].

The role of inflammation in cancer is dual, involving both local and systemic aspects. Local inflammation represents a local immune response mediated by cytokines, chemokines, and other small inflammatory proteins originating from tumor stromal cells or host immune cells [27]. Tumor necrosis, which promotes tumor growth, is also significant in this process [28]. Two possible explanations exist for the association between tumor necrosis and inflammatory infiltrate. The first theory suggests that tumor necrosis occurs due to a high proliferation rate of tumor cells, which outgrow the existing oxygen supply, leading to hypoxia-induced cell death. The second theory proposes that tumor cells, through their proliferation, stimulate increased production of cytokines and chemokines, which attract immune cells that induce tumor cell apoptosis, thus resulting in tumor necrosis [26,29].

Systemic inflammation consists of circulating cytokines, small inflammatory proteins, circulating immune cells (leukocytes, monocytes, dendritic cells, platelets), and acute-phase proteins (CRP, amyloid A, alpha-1 antitrypsin). These inflammatory mediators lead to the development of clinical symptoms that represent the presence and progression of cancer [27]. Cytokines and chemokines contribute to tumor dissemination to distant body sites and the occurrence of distant metastases. The clinical picture and course of the disease are affected by the clinical manifestations of systemic inflammation and the neoplastic process. Clinical manifestations include so-called B symptoms—fever, night sweats, weight loss exceeding 5%, fatigue, malnutrition, and malignant cachexia [29].

A retrospective cohort study by Kaiming Zhang et al., published in 2021 [30], investigated the prognosis of BC patients with a systemic inflammatory response (SIRS). It has shown that patients with high SIRS scores had significantly worse disease prognoses than those with low SIRS values. The results also demonstrated a significant association between SIRS and tumor multifocality (*p* = 0.004) and axillary lymph node status (*p* = 0.017). In contrast, no significant associations were found with tumor size (*p* = 0.187), age (*p* = 0.072), vascular cancer emboli (VCE), or histological grade (0.588). In the T1/2 (*p* = 0.00019) and T3/4 (*p* = 0.048) groups, there was a statistically significant difference in overall survival (OS) between low and high SIRS values. Furthermore, in patients with positive axillary lymph nodes, OS was better in those with low SIRS values than in those with high values (*p* < 0.0001). In contrast, in the group without lymph node metastases, there was no difference in OS regardless of SIRS value (*p* = 0.069). Univariate analysis showed that age (*p* = 0.003), SIRS (*p* < 0.001), VCE (*p* < 0.001), size of the tumor (*p* < 0.001), N stage (*p* < 0.001), and pathological classification (*p* < 0.001) were prognostic indicators for OS in BC patients and that multifocality of cancer had no prognostic significance (*p* = 0.340). Multivariate analysis showed that age (*p* = 0.014), SIRS (*p* < 0.001), VCE (*p* = 0.039), size of the tumor (*p* = 0.021), N stage (*p* < 0.001) and pathological classification (*p* < 0.001) were independent prognostic factors for BC patients [30].

A prospective nested case–control study conducted by Nicholas P. McAndrew et al. in 2021 [31] on 1287 BC patients investigated the impact of inflammation on the relapse in early BC treated with aromatase inhibitors (AIs) in the adjuvant setting. Estrogen-positive breast cancer accounts for 60–80% of all invasive breast cancers. In the adjuvant treatment of this type of cancer, AIs block the conversion of androgens to estrogen, with the target site of action being adipose tissue. AI therapy has improved survival and reduced recurrence rates in estrogen-positive BC. However, about 30% of patients experience tumor recurrence. The reasons for recurrence in some women are still unknown, but one hypothesis is that inflammation plays a significant role. Patients were divided into two cohorts based on HER2 status. CRP (*p* = 0.019) and SAA (*p* = 0.002) levels were independently associated with an increased risk of relapse in the ER+/HER2− group, while no such association was found in the ER+/HER2+ group (for CRP *p* = 0.5423; for SAA *p* = 0.8337). Levels of IL-6 were greater than or equal to the median value and were not associated with an increased risk of relapsed BC in both cohorts (for ER+/Her2− *p* = 0.367; for ER+/Her2+ *p* = 0.3617). The mechanisms of these processes are still unknown [31]. Table 2 presents the results of the studies discussed in this section.

### 3.3. Dyslipidemia

Dyslipidemia includes high total triglyceride (TG) levels, high total cholesterol (TC) levels, and low serum HDL levels. Dyslipidemia is considered to be associated with the development of BC. Elevated cholesterol levels are a well-established risk factor for coronary artery disease and stroke, while their role in carcinogenesis is not fully understood [32]. The review article by Dong S. from 2021 stated that lipid disorders are a risk factor for BC. However, the relationship between different serum lipids and the risk of different subtypes of breast cancer still needs to be confirmed through more clinical studies [5].

The retrospective study by Youzhao Ma et al. from 2023 [33] investigated the association of dyslipidemia with poor prognosis in patients receiving neoadjuvant chemotherapy. The results indicated that patients with dyslipidemia were older and had a higher BMI. There was a significant increase in TG levels (*p* < 0.001), TC levels (*p* < 0.001), and low-density lipoprotein cholesterol (LDL-C) levels (*p* < 0.001), as well as a significant decrease in high-density lipoprotein cholesterol (HDL-C) levels (*p* < 0.001), after chemotherapy. Furthermore, it was shown that TG (*p* = 0.04) and LDL-C (*p* = 0.028) levels were significantly increased in premenopausal patients. In contrast, HDL-C levels (*p* < 0.001) were significantly decreased, with no statistically significant change in TC levels. In postmenopausal patients, chemotherapy led to elevated levels of TC, TG, and LDL-C and reduced levels of HDL-C (*p* < 0.001). Preoperative lipid levels were also significantly associated with axillary pCR rates (*p* < 0.05), with the rates of axillary pCR being significantly higher in patients with normal lipid profiles compared to those with dyslipidemia (*p* = 0.009) [33].

Prospective studies investigating the relationship between lipid metabolism and breast cancer have shown inconsistent results. The study by Cari M. Kithara et al. from 2011 [32] showed that high TC levels were positively associated with the risk of BC in women (*p* = 0.03). After excluding the first five years of follow-up, the association with BC risk was stronger (*p* = 0.003) [32]. A prospective study conducted by Mathilde His et al. in 2013 [34] examined the association between TC, HDL-C, LDL-C, apolipoprotein A1 (apoA1), B, and TG and the risk of developing breast cancer and prostate cancer. The results indicated that serum levels of TC (*p* = 0.04), HDL-C (*p* = 0.009), and apoA1 (*p* = 0.004) were associated with reduced overall risk of breast cancer. In contrast, the values of the LDL-C/HDL-C ratio (*p* = 0.049) and TC/HDL-C ratio (*p* = 0.03) were associated with an increased risk [34].

In a prospective case–control study conducted by Poonam Kachhawa et al. in 2018 [35], the aim was to compare the levels of serum lipids in patients with BC to healthy controls and to determine the influence of dyslipidemia and insulin resistance on the risk of breast cancer occurrence. The study showed that women with BC had elevated levels of TC, TG, LDL-C, VLDL-C, fasting glucose, fasting serum insulin, and HOMA-IR compared to healthy controls. Additionally, TGs were significantly elevated in patients with both pre- and postmenopausal BC compared to healthy controls. There was no statistically significant difference between pre- and postmenopausal BC women and controls in HDL-C values. This study concluded that dyslipidemia and impaired glucose metabolism are significantly associated with BC. Furthermore, the authors found that levels of TC, LDL-C, TG, and serum glucose are important risk factors in the development of BC [35]. The findings from the studies discussed in this paragraph are summarized in Table 3.

### 3.4. Obesity

Obesity is a chronic metabolic disorder of multifactorial origin, with its prevalence continuously increasing [36]. In adults, obesity is defined as a body mass index (BMI) equal to or greater than 30 kg/m2, while overweight is represented by a BMI greater than 25 kg/m^2^. Health risks that arise from obesity include various diseases such as hypertension, type 2 diabetes mellitus, dyslipidemia, cardiovascular diseases, and several types of cancer [37]. Obesity is a preventable risk factor for the development of BC [38]. Abdominal obesity promotes cardiovascular risk factors such as glucose, lipid disorders, and elevated blood pressure [39]. Previous studies have indicated a positive correlation between obesity and breast cancer in postmenopausal patients and a negative correlation in premenopausal patients. Recent studies utilizing both BMI and waist–hip ratio (WHR) demonstrate that obesity is associated with increased morbidity and mortality in both pre- and postmenopausal patients [40].

Obesity in insulin resistance is a chronic inflammatory condition with local and systemic manifestations [41]. It has been shown that obese individuals with insulin resistance have abnormal levels of adipokines in the serum—reduced levels of adiponectin and elevated levels of leptin—which are also associated with an increased risk of obesity-related cancer development [42,43].

In the meta-analysis conducted by Melinda Protani et al. in 2010 [44], 43 studies were analyzed. Research showed that survival among obese women with breast cancer was worse than survival among non-obese women (HR = 1.33). The study also suggested that obesity might be a stronger determinant of survival in premenopausal than postmenopausal women. This difference was not statistically significant, and the authors suggested that more studies are needed to prove this difference [44].

In a prospective cohort study conducted by Eugenia E. Calle et al. in 2003 [45] involving 900,000 individuals without a cancer diagnosis at the time of study initiation, over a 16-year follow-up period, 57,145 deaths occurred as a result of malignancies. This study investigated the association between BMI and the risk of death due to malignancies. The results indicated that elevated BMI was correlated with fatal outcomes across various types of malignancies. The study concluded that implementing interventions to maintain a normal body mass could potentially prevent 90,000 cancer-related deaths annually [45].

The descriptive cross-sectional study by Marianne Ewertz et al. from 2011 investigated the impact of obesity on the risk of breast cancer recurrence and death as a result of BC or other causes in relation to adjuvant treatment. The study included 53,816 women with early-stage BC. Table 4 presents prognostic characteristics according to BMI values from this research.

The median follow-up period was 7.1 years, and in this period, there were 4180 locoregional recurrences and 7278 distal metastases. BMI had no impact on the risk of locoregional recurrences, but there was an increased risk of distal metastases with increasing BMI. The authors found that the risk of distant metastases changes with time since diagnosis; for the first 5 years after diagnosis, there was no association with BMI, but from 5 to 10 years after diagnosis, the risk of developing distant metastases increased significantly—by 42% to 46% for patients with BMI of 25 kg/m^2^ or greater as compared with patients with a BMI less than 25 kg/m^2^. At the end of the follow-up, 15,197 patients had died as a result of breast cancer, and 5967 had died from other and unknown causes. There was a trend of increasing risk of dying as a result of breast cancer with increasing BMI. Results also showed that adjuvant therapy, both chemo- and endocrine therapy, was less effective in patients with a BMI of 30 kg/m^2^ or greater after ten years of follow-up. The study concluded that obesity is an independent prognostic factor for the occurrence of distant metastases and fatal outcomes in BC patients but not for locoregional recurrences. Additionally, the study suggested that adjuvant chemotherapy is less effective in BC patients who are obese. Studies indicate that a healthy diet with a high intake of fruits, vegetables, whole grains, and low-fat products may be associated with better outcomes. However, interventional studies have not confirmed this hypothesis, and there is no evidence that supplementation with vitamins and minerals improves outcomes [46]. A summary of results from the studies discussed in this paragraph is provided in Table 5.

### 3.5. Diabetes

Diabetes is associated with an increased risk of developing various diseases, including cardiovascular disease (CVD). Research also indicates an elevated risk of cancer associated with diabetes [47].

Diabetes mellitus is one of the most prevalent chronic diseases worldwide. It is characterized by hyperglycemia. Dysfunction of the pancreatic insulin-producing B cells and insulin resistance mainly lead to hyperglycemia [48].

A review article by Samson Mathews Samuel et al. from 2018 [47] compared several meta-analyses linking DM2 to increased incidence of BC and poorer response to systemic therapy. It has been shown that female patients with DM2 have a worse prognosis compared to those without DM, indicating a connection between DM2 and cancer progression [47,49].

Previous studies suggested that there is no association between type 1 diabetes and breast cancer incidence. It is considered that hyperglycemia and insulin resistance, which are present in DM2, represent the risk factors for breast cancer development, leading to increased mortality regardless of menopausal status [50,51].

In a retrospective cohort study conducted by Michael G. Schrauder et al. in 2010 [52], it was demonstrated that mortality among breast cancer patients is significantly higher in those with DM compared to those without DM. The increased risk associated with DM did not show statistical significance between pre- and postmenopausal women. DM was found to be a significant independent predictor of mortality in BC patients. DM2 showed no significant association with local recurrence-free survival and distant metastasis-free survival in the whole cohort. However, a significant association of DM with a higher distant metastasis rate during the five-year follow-up period was found only in the estrogen receptor (ER)-negative subgroup [52].

In the prospective cohort study by Fanxiu Xiong et al., published in 2023 [53], no association was found between diabetes and BC risk overall. The results indicated that women with type 1 diabetes or those recently diagnosed with type 2 diabetes may have an increased risk of developing BC. These findings differ from those previously mentioned, which claim no association between type 1 diabetes and breast cancer [53].

A case–cohort study by Rola Hamood et al. from 2018 [54] investigated the risk of developing DM in patients receiving hormonal therapy (HT) in the adjuvant setting. In contrast to studies examining DM as a risk factor for breast cancer, this study examined whether women who had survived breast cancer and were receiving HT were at increased risk of developing DM. The study showed that women receiving HT are at increased risk of developing DM, but also suggested that the study should be repeated with a larger sample size. It was also noted that these results do not imply that HT should be discontinued. However, consideration should be given to addressing other modifiable factors, such as lifestyle changes and introducing screening tests for early detection of DM [54]. The studies mentioned in this section are summarized in Table 6.

## 4. Conclusions

Numerous studies have been conducted investigating cardiovascular risk, metabolic syndrome, inflammation, dyslipidemia, obesity, and diabetes in patients with breast cancer. Despite the extensive analyses, the pathophysiological mechanisms of these associations still need clarification. Premenopausal breast cancer patients have not been thoroughly investigated, while much attention has been devoted to postmenopausal breast cancer patients. Previous research has not proposed potential health interventions. Multicenter prospective clinical studies are needed to establish the precise association and pathophysiological mechanisms linking cardiovascular risk, metabolic syndrome, obesity, diabetes, and inflammation in breast cancer patients.

## Figures and Tables

**Table 1:** Preview of the studies included in Section 3.1.

Study, Author, Year	Type of Study	Objective	Number of Patients	Results
Oxidative stress in normal-weight obese syndrome, Laura Di Renzo et al., 2010 [15]	Prospective	To verify if early inflammation is accompanied by oxidative stress in NWO women	60	Significant difference between BMI-NW and NOW, NW and OB (*p* < 0.05). Correlation analysis revealed strong associations between GSH levels and BW, BMI (R = −0.45; *p* < 0.05), waist circumference (R = −0.33, *p* < 0.05), and Tg (R = −0.416, *p* < 0.05); LOOH levels were negatively related to FFM% (R = −0.413, *p* < 0.05) and positively to FM%, IL-15, TNF-α, insulin, total cholesterol, LDLH and TG (R = 0.408, R = 0.502, R = 0.341, R = 0.412, R = 0.4036, R = 405, R = 0.405, *p* < 0.05).
Normal-weight obese syndrome: Early inflammation? Antonino De Lorenzo et al., 2007 [16]	Prospective	To define the relations between anthropometric variables, lipid indexes, and secretion of proinflammatory cytokines as significant prognostic indicators of CVD risk and MetS	60	Plasma values and body composition measures were significantly different between preobese–obese and non-obese women. No significant differences in body weight, laboratory values or CVD risk factors were found between the NWO and non-obese groups. Plasma concentrations of IL-1α, IL-1β, IL-6, IL-8, and TNF-α were significantly lower in non-obese group and significantly greater in preobese–obese group compared to NWO (*p* < 0.05).
Diet quality and mortality risk in metabolically obese normal-weight adults, Yong-Moon Mark Park et al., 2016 [14]	Prospective	To examine the associations among the Dietary Approaches to Stop Hypertension-style diet, the Healthy Eating Index, and metabolic risk in MONW	2103	MONW adherence to DASH diet (17% [HR 0.83; 95% CI, 0.72–0.97]) or HEI (22% [HR 0.78, 95% CI, 0.68–0.90] was significantly associated with reductions in the risk of all-cause mortality. Reduction in cancer mortality with 1-SD increment of HEI (HR 0.63; 95% CI, 0.46–0.88). No association in MHNW phenotype.
Breast cancer risk in metabolically healthy but overweight postmenopausal women, Mark J. Gunter et al., 2015 [18]	Retrospective	To compare the risk of incident postmenopausal BC among MHOW and MHNW patients	2830	Metabolically healthy overweight women, defined using HOMA-IR, were not at elevated risk of BC compared to MHNW (HR_HOMA-IR_ = 0.96; 95% CI, 0.64–1.42). Risk among women with high HOMA-IR was elevated whether they were overweight (HR_HOMA-IR_ = 1.76; 95% CI, 1.19–2.60) or normal-weight (HR_HOMA-IR_ = 1.80; 95% CI 0.88–3.7). Using fasting insulin to define metabolic health, metabolically unhealthy women were at higher risk of breast cancer regardless of whether they were normal-weight (HR_insulin_ = 2.96; 95% CI 1.01–4.22) or overweight (HR_insulin_ = 2.01; 95% CI 1.35–2.99). Metabolically healthy overweight women did not have significantly increased risk of BC compared to MHNW (HR_insulin_ = 0.96; 95% CI 0.64–1.42).
The association between Metabolic Health, obesity phenotype and the risk of breast cancer, Yong-Moon Mark Park et al., 2017 [19]	Prospective	To examine whether the risk of BC differs by metabolic status among those in the same category of BMI	43,599	Women with BMI < 25 kg/m^2^ and ≥1 metabolic abnormality had increased risk of postmenopausal BC (HR = 1.26 95% CI: 1.01–1.56) Women with BMI ≥ 25 kg/m^2^ and no metabolic abnormalities had increased risk of postmenopausal BC (HR = 1.24, 95% CI: 0.99–1.55). Risk of postmenopausal BC elevated in women with normal BMI and central obesity regardless of criterion used to define central obesity.
Metabolic health reduces risk of obesity-related cancer in Framingham study adults, Lynn L. Moore et al., 2014 [23]	Prospective	To estimate the risk of obesity-related cancers among overweight/obese individuals according to their metabolic health	3763	Overweight women with elevated blood glucose had a 2.6-fold increased risk (95% CI: 1.4–4.9) of female reproductive cancers and postmenopausal BC. Overweight women with normal glucose levels had 70% increased risk (95% CI: 1.1–2.5).
The long-term prognosis of cardiovascular disease and all-cause mortality for metabolically healthy obesity: A systematic review and meta-analysis. Ruizhi Zheng et al., 2016 [20]	Systematic review and Meta-analysis	To assess the risks of cardiovascular events and all-cause mortality for MHO individuals	584,799	Association between the MHO phenotype and the risk of CV events. RR and HR 1.50 (95% CI 1.27 to 1.77) and 1.60 (95% CI 1.38 to 1.84). Risk of all-cause mortality associated with the MHO phenotype. For unadjusted dataset: RR 1.18 (95% CI 0.83 to 1.66, I^2^ = 84.5%, *p* < 0.001 for heterogeneity). For adjusted dataset: 1.07 (95% CI 0.92 to 1.25, I^2^ = 17.6%, *p* = 0.276 for heterogeneity).

**Table 2:** Preview of the studies included in Section 3.2.

Study, Author, Year	Type of Study	Objective	Number of Patients	Results
A systemic inflammation response score for prognostic prediction of breast cancer patients undergoing surgery, Kaiming Zhang et al., 2021 [30]	Retrospective	To explore the prognostic value of systemic inflammation	1583	SIRS was an independent prognostic factor; high SIRS is related to multifocality, advanced N stage, and worse prognosis.
Effects of systemic inflammation on relapse in early breast cancer, Nicholas P. McAndrew, 2021 [31]	Prospective	To examine if patients with high circulating levels of inflammatory cytokines and high-risk IL-6 promoter genotypes are more likely to recur during or after AI treatment compared to those without elevated inflammatory markers	185	Cases had significantly higher median serum levels of CRP relative to controls (9.54 vs. 3.25 mg/L, *p* = 0.004) and SAA (11.03 vs. 6.81 mg/L, *p* = 0.009); serum IL-6 concentrations did not differ in controls *p* = 0.7911. Serum CRP or SAA level ≥ the median value was significantly associated with breast cancer relapse; CRP OR 2.4 (95% CI 1.16–5.00) and SAA OR 3.38 (95% CI 1.57–7.25, *p* = 0.002). Increasing CRP and SAA levels were associated with a significantly increased risk of relapse. For CRP OR 1.68 (95% CI 1.25–2.26, *p* = 0.001). For SAA OR 1.79 (95% CI 1.18–2.72, *p* = 0.007). IL-6 levels were not associated with increased risk of BC relapse (OR 0.98, 95% CI 0.64–1.52, *p* = 0.940).
The systemic inflammatory response and its relationship to pain and other symptoms in advanced cancer, Barry J. Laird et al., 2013 [27]	Retrospective	To examine the relationship between symptoms and systemic inflammation in a large cohort of patients with advanced cancer	1466	Performance status (*p* = 0.179), survival (*p* = 0.347), pain (*p* = 0.154), anorexia (*p* = 0.206), cognitive dysfunction (*p* = 0.137), dyspnea (*p* = 0.150), fatigue (*p* = 0.197), physical dysfunction (*p* = 0.132) and poor quality of life (*p* = 0.178) were associated with increasing levels of systemic inflammation with *p* < 0.001.

**Table 3:** Preview of the studies included in Section 3.3.

Study, Author, Year	Type of Study	Objective	Number of Patients	Results
Dyslipidemia is associated with a poor prognosis of breast cancer in patients receiving neoadjuvant chemotherapy, Youzhao Ma et al., 2023 [33]	Retrospective	To examine the effects of NAHT on serum lipid level, the correlation between serum lipid level and clinicopathological features, and the effect of the serum lipid level on pCR and DFS	312	The baseline serum lipid level was significantly correlated with their age and BMI (*p* < 0.05). Chemotherapy increased the levels of TG, TC and LDL, but decreased the level of HDL (*p* < 0.001). Preoperative dyslipidemia was significantly associated with the axillary pCR rate (*p* < 0.05). Prognostic factors affecting DFS in BC are full-course serum lipid level (HR = 1.896 [95% CI 1.609–3.360] *p* = 0.029), N stage (HR = 4.416 [95% CI 2.348–8.308]; *p* < 0.001), and the total pCR rate (HR = 4.319 [95% CI 1.029–18.135]; *p* = 0.046). The relapse rate in patients with a high level of TC was higher than that in patients with a high level of TG (61.9% vs. 30.0%; *p* < 0.05).
Total cholesterol and cancer risk in a large prospective study in Korea, Cari M. Kithara et al., 2011 [32]	Prospective	To examine the association between TC and risk of all and site-specific cancer incidence	1,189,719	High TC (≥240 mg/dL) was positively associated with BC in women (HR = 1.17; 95% CI, 1.03–1.33; *p* trend= 0.03). TC was inversely associated with all-cancer incidence in both men (HR, 0.84; 95% CI, 0.81–0.86, *p* trend < 0.001) and women (HR, 0.91; 95% CI, 0.87–0.95; *p* trend < 0.001), but these associations were attenuated after excluding incident liver cancers (men: HR 0.95, *p* trend < 0.001; women: HR, 0.98; *p* trend = 0.32).
Prospective associations between serum biomarkers of lipid metabolism and overall, breast and prostate cancer risk, Mathilde His et al., 2014 [34]	Prospective	To investigate the association between TC, HDL-C, LDL-C, apoA1, apoB, TG, and overall BC and prostate cancer risk	7557	Inverse associations with breast cancer risk: TC (HR = 0.83, 95% CI 0.69–0.99; *p* = 0.04). HDL-C (HR = 0.48, 95% CI 0.28–0.83; *p* = 0.009). apoA1 (HR = 0.36, 95% CI 0.18–0.73; *p* = 0.004).
Association of dyslipidemia, increased insulin resistance, and serum ca 15-3 with increased risk of breast cancer in urban areas of North and Central India, Poonam Kachhawa et al., 2018 [35]	Prospective	To compare serum lipid levels in female BC patients with those in normal healthy controls and to discover the effect of dyslipidemia and increased IR on BC	253	TC, TG, LDL, VLDL, serum glucose, serum insulin, HOMA-IR, and serum CA 15-3 were significantly higher (*p* < 0.001) in BC patients. Significant ORs with 95% CI were serum glucose, TC and TG. Significant positive correlation between TC, TG, LDL, serum glucose, serum insulin, HOMA-IR and serum CA 15-3.

**Table 4:** Prognostic characteristics according to BMI values from the study “Effect of Obesity on Prognosis After Early-Stage Breast Cancer” by Marianne Ewertz.

Characteristic	BMI > 30 kg/m^2^ in Comparison with BMI < 25 kg/m^2^	*p*-Value
Age	Older	*p* < 0.001
Menopausal status	More often postmenopausal	*p* < 0.001
Tumor size	Larger tumor size	*p* < 0.01
Removed nodes	More lymph nodes removed	*p* < 0.001
Positive nodes	More lymph nodes positive	*p* < 0.001
Fascial invasion	Less deep fascia invasion	*p* < 0.001
Histological type and grade	More ductal grade 3	*p* = 0.04
ER status	No significant difference	*p* = 0.4

**Table 5:** Preview of the studies included in Section 3.4.

Study, Author, Year	Type of Study	Objective	Number of Patients	Results
Effect of obesity on survival of women with breast cancer: Systematic Review and meta-analysis, Melinda Protani et al., 2010 [44]	Systematic review and meta-analysis	To examine whether the women who were obese at the time of diagnosis of invasive BC had worse overall or BC-specific survival than non-obese women	Median 1192	Poorer survival among obese women with BC. Overall survival (HR = 1.33; 95% CI 1.21–1.47). BC-specific survival (HR = 1.33; 95% CI 1.19–1.50).
Overweight, obesity, and mortality from cancer in a prospectively studied cohort of U.S. adults, Eugenia E. Calle et al., 2003 [45]	Prospective	To determine the relations between BMI and the risk of death from cancer at specific sites	900,053	Significant trends of increasing risk with higher BMI values for death from cancer of the breast, uterus, cervix, and ovary in women. Relative risk of death for women 1.62 (95% CI: 1.40–1.87).
Effect of obesity on prognosis after early-stage breast cancer, Marianne Ewertz et al., 2011 [46]	Retrospective	To characterize the impact of obesity on the risk of BC recurrence and death as a result of BC or other causes in relation to adjuvant treatment	53,816	Patients with BC and BMI ≥ 30 kg/m^2^ were older (*p* < 0.001), more often postmenopausal (*p* < 0.001), had larger tumors (*p* < 0.001), and had more advanced disease at diagnosis compared to patients with BMI < 25 kg/m^2^ (*p* < 0.001). For patients with BMI ≥ 30 kg/m^2^ risk of developing distant metastases after 10 years was significantly increased by 46% and the risk of dying as a result of BC after 30 years was significantly increased by 38%.
Comorbidity associated with obesity in a large population: The Apna study, Elena Martin-Rodriguez et al., 2015 [36]	Retrospective	To estimate the comorbidity associated with obesity	40,010	Increased BMI is associated with glucose intolerance (OR: 1.07; 95% CI 1.06–1.08), dyslipidemia (OR: 1.04; 95% CI 1.03–1.04), hypertension (OR: 1.12; 95% CI 1.12–1.13), type 2 diabetes (OR: 1.11; 95% CI 1.10–1.11), kidney failure (OR: 1.04; 95% CI 1.03–1.05) and osteoarthritis (OR: 1.06; 95% CI 1.05–1.06)
Circulating levels of MCP-1 and IL-8 are elevated in human obese subjects and associated with obesity-related parameters, C-S Kim et al., 2006 [41]	Prospective	To examine association between circulating levels of selected chemokines, obesity-related parameters and CRP	100	MCP-1 and IL-8 in the serum were significantly higher (*p* < 0.05) in obese subjects (BMI > 30 kg/m^2^). The levels of CRP were positively correlated with BMI (*p* < 0.001) or waist circumference (*p* < 0.0001). The levels of MCP-1 and IL-8 were positively correlated with BMI (MCP 1 *p* < 0.02; IL-8 *p* < 0.01) and/or waist circumference (MCP 1 *p* < 0.009; IL-8 *p* < 0.03). The levels of MCP-1 were positively related to the levels of CRP (*p* < 0.007) or interleukin-6 (*p* < 0.0001), and negatively related to the levels of HDL-cholesterol (*p* < 0.01).

**Table 6:** Preview of the studies included in Section 3.5.

Study, Author, Year	Type of Study	Objective	Number of Patients	Results
Diabetes and prognosis in a breast cancer cohort, Michael G. Schrauder et al., 2011 [52]	Retrospective	To examine influence of DM on survival, distant metastasis-free survival and local recurrence-free survival in relation to common tumor and patient characteristics	4056	Women with DM were significantly older, had larger tumors, and a higher rate of lymph node involvement. After a follow-up period of 5 years, overall mortality following BC was significantly higher in diabetic BC patients (HR 1.92, 95% CI 1.49–2.48). There were no significant differences in distant metastasis-free survival (HR 1.10; 95% CI 0.74–1.64) and local recurrence-free survival (HR 0.82; 95% CI 0.45–1.48). Slightly significantly higher rate of distant metastasis in the group of patients with DM and ER-negative tumors (HR 2.28; 95% CI 1.31–3.97).
Diabetes mellitus and risk of breast cancer: A large-scale, prospective, population-based study, Fanxiu Xiong et al., 2022 [53]	Prospective	To examine associations of diabetes overall, T1D, and T2D with risk of incident BC	250,312	No overall association between DM and BC risk (HR = 1.02, 95% CI 0.92–1.14). Women with T1D had a higher risk of BC than women without diabetes (HR = 1.52, 95% CI 1.03–2.23). T2D was not associated with BC risk overall (HR 1, 95% CI 0.90–1.12).
Diabetes after hormone therapy in breast cancer survivors: A case–cohort study, Rola Hamood et al., 2018 [54]	Retrospective	To examine the association between hormone therapy and diabetes risk in BC survivors	2246	The crude cumulative incidence of diabetes that accounted for death as a competing risk was 20.9% (95% CI 18.3–23.7%). Hormone therapy was associated with increased diabetes risk (HR 2.40; 95% CI 1.26–4.55; *p* = 0.008). The hazard for tamoxifen use (HR 2.25; 95% CI 1.19–4.26; *p* = 0.013) was less pronounced than the use of aromatase inhibitors (HR 4.27, 95% CI 1.42–12.84; *p* = 0.010).
Metformin and thiazolidinediones are associated with improved breast cancer-specific survival of diabetic women with her2+ breast cancer, X. He et al., 2011 [49]	Retrospective	To examine the impact of different classes of antidiabetic pharmacotherapy on breast cancer-specific survival.	1983	DM2 predicted poor survival of stage ≥ HER2+ BC (*p* = 0.026, HR 1.42, 95% CI 1.04–1.94). Metformin and thiazolidinediones predicted lengthened survival (*p* = 0.041, HR 0.52, 95% CI 0.28–0.97; *p* = 0.036; HR 0.41, 95% CI 0.18–0.93, respectively). In the DM group metformin and thiazolidinediones predicted longer survival and were associated with decreased BC-specific mortality (*p* = 0.023, HR 0.47, 95% CI 0.24–0.90; *p* = 0.044; HR 0.42, 95% CI 0.18–0.98 respectively).
Cancer and diabetes—A follow-up study of two population-based cohorts of diabetic patients, H. Hjalgrim et al., 1997 [51]	Retrospective	To examine the risk of cancer amongst patients with insulin-treated DM	3158	No unusual risk of cancer was observed among the conscripts or among patients with onset of DM before the age of 39 years.

## Data Availability

No new data were created or analyzed in this study. Data sharing is not applicable to this article.
